# Higher FORTA (Fit fOR The Aged) scores are associated with poor functional outcomes, dementia, and mortality in older people

**DOI:** 10.1007/s00228-022-03389-w

**Published:** 2022-09-27

**Authors:** Farhad Pazan, Hanna Breunig, Christel Weiss, Susanne Röhr, Melanie Luppa, Michael Pentzek, Horst Bickel, Dagmar Weeg, Siegfried Weyerer, Birgitt Wiese, Hans-Helmut König, Christian Brettschneider, Kathrin Heser, Wolfgang Maier, Martin Scherer, Steffi Riedel-Heller, Michael Wagner, Martin Wehling

**Affiliations:** 1 Clinical Pharmacology Mannheim, Medical Faculty Mannheim, Ruprecht-Karls-Heidelberg University, Theodor-Kutzer-Ufer 1-3, 68167 Mannheim, Germany; 2 Department of Medical Statistics, Biomathematics and Information Processing, Medical Faculty, University of Heidelberg, Mannheim, Germany; 3 Institute of Social Medicine, Medical Faculty, Occupational Health and Public Health (ISAP), University of Leipzig, Leipzig, Germany; 4 Institute of General Practice, Medical Faculty, Heinrich-Heine-University Düsseldorf, Düsseldorf, Germany; 5 Department of Psychiatry, Technical University of Munich, Munich, Germany; 6 Medical Faculty Mannheim, Central Institute of Mental Health, Heidelberg University, Mannheim, Germany; 7 Institute for General Practice, Hannover Medical School, Hanover, Germany; 8 Department of Health Economics and Health Services Research, University Medical Centre Hamburg-Eppendorf, Hamburg, Germany; 9 Department of Neurodegenerative Diseases and Geriatric Psychiatry, University Hospital Bonn, Bonn, Germany; 10 Department of Primary Medical Care, Center for Psychosocial Medicine, University Medical Center Hamburg-Eppendorf, Hamburg, Germany; 11 German Center for Neurodegenerative Diseases (DZNE), Bonn, Germany

**Keywords:** Polypharmacy, Multimorbidity, Inappropriate prescribing, Dementia, Older people

## Abstract

**Purpose:**

Higher Fit fOR The Aged (FORTA) scores have been shown to be negatively associated with adverse clinical outcomes in older hospitalized patients. This has not been evaluated in other health care settings. The aim of this study was to examine the association of the FORTA score with relevant outcomes in the prospective AgeCoDe–AgeQualiDe cohort of community-dwelling older people. In particular, the longitudinal relation between the FORTA score and mortality and the incidence of dementia was evaluated.

**Methods:**

Univariate and multivariate correlations between the FORTA score and activities of daily living (ADL) or instrumental activities of daily living (IADL) as well as comparisons between high vs. low FORTA scores were conducted.

**Results:**

The FORTA score was significantly correlated with ADL/IADL at baseline and at all follow-up visits (*p* < 0.0001). ADL/IADL results of participants with a low FORTA score were significantly better than in those with high FORTA scores (*p* < 0.0001). The FORTA score was also significantly (*p* < 0.0001) correlated with ADL/IADL in the multivariate analysis. Moreover, the mean FORTA scores of participants with dementia were significantly higher (*p* < 0.0001) than in those without dementia at follow-up visits 6 through 9. The mean FORTA scores of participants who died were significantly higher than those of survivors at follow-up visits 7 (*p* < 0.05), 8 (*p* < 0.001), and 9 (*p* < 0.001).

**Conclusion:**

In this study, an association between higher FORTA scores and ADL as well as IADL was demonstrated in community-dwelling older adults. Besides, higher FORTA scores appear to be linked to a higher incidence of dementia and even mortality.

**Supplementary Information:**

The online version contains supplementary material available at 10.1007/s00228-022-03389-w.

## Introduction

Globally, the number as well as the share of older people in the total population has been constantly growing and this trend is expected to continue in the future [1, 2]. Consequently, as the prevalence of multimorbidity (having two or more chronic diseases) and associated polypharmacy (use of five or more medications by most definitions) is generally high in this group [3–5], these issues are expected to become even more relevant over the coming decades. Polypharmacy often leads to inappropriate drug treatment and this frequently results in preventable adverse clinical outcomes [2, 6–16]. Inappropriate prescribing is often the result of an “evidence-biased” pharmacotherapy in older adults [17]. This “evidence-biased” or “age-blind” [17] pharmacotherapy is based on a lack of evidence on benefits and risks of many medications in older people [16], who are often excluded from clinical trials [18]. To address this problem, numerous listing approaches/criteria combining the available evidence with experts’ opinion have been developed [19–24].

Most of these listing approaches do not require elaborate patient knowledge and mainly focus on de-prescribing, e.g., the Beers Criteria®. However, few of them require intricate knowledge regarding diagnoses, severity, functionality, and patient’s wishes/needs and therefore aid physicians in addressing the problem of undertreatment as well as overtreatment. These decisive differences between the available tools led us to a more precise categorization of these tools into either drug-oriented listing approaches (DOLA) or patient-in-focus listing approaches (PILA) [21]. For instance, the Beers Criteria®, which mainly represent a negative-list of medications, is a DOLA. In contrast, the Screening Tool to Alert Doctors to the Right Treatment (START)/Screening Tool of Older Persons’ Prescriptions (STOPP) criteria [20] or the Fit fOR The Aged (FORTA) list [23], which combine positive and negative labeling of drug treatments [16], are PILAs [21]. Based on rare randomized controlled trials, a positive impact of DOLAs on clinical outcomes is largely missing, as opposed to the consistent improvement of such endpoints by PILAs [21].

As a PILA, FORTA has been validated in a randomized controlled trial (VALFORTA) [25] in older hospitalized patients. This trial showed that the use of FORTA significantly improves the quality of medication as measured by the FORTA score reflecting the sum of over- and undertreatment prescription errors according to the FORTA list. In addition, several clinical outcomes such as adverse drug reactions or the activities of daily living (ADL) were significantly improved by the FORTA intervention. In a cross-sectional secondary analysis of the VALFORTA trial [16], an association of the FORTA score as a measure of medication quality with adverse outcomes such as impaired instrumental activities of daily living (IADL) or Mini-Mental State Examination (MMSE) has been shown as well.

The aim of this study was to conduct a cross-sectional and a longitudinal analysis of “The Study on Ageing, Cognition and Dementia”– “The Study on Needs, health service use, costs and health-related quality of life in a large sample of oldest-old primary care patients” (AgeCoDe–AgeQualiDe) prospective cohort study in community-dwelling older patients [26–29] to corroborate the previously observed association of the FORTA score [16, 25] with relevant functional outcomes. In addition to the findings for older multimorbid hospitalized patients from the VALFORTA trial, here a larger cohort of older outpatients was examined by the assessment of functional outcomes, mortality and the incidence of dementia.

## Methods

### Study design

The AgeCoDe study is a German multi-centered (Bonn, Düsseldorf, Hamburg, Leipzig, Mannheim and Munich) population-based longitudinal cohort study, which started in 2003/2004. For this study, primary care patients aged 75 years and older who had no dementia at baseline were recruited via general practitioners’ (GP) offices. Patients needed to have had at least one contact with the GP during the preceding 12 months [30]. In total, 3327 patients [31] were investigated and 3214 patients were included in this study at baseline; 6 follow-up visits were recorded. Follow-up assessments took place every 1.5 years on average [27] and the 6^th^ follow-up concluded in January 2014 [27]. Trained psychologists and physicians visited GP patients at home and conducted structured clinical interviews [27]. The AgeCoDe study was later extended by the AgeQualiDe study (follow-up visits 7 to 9). In AgeQualiDe, the time span between each follow-up was 10 months [32]. The data collection for follow-up 7 occurred between January 2014 and September 2015 [33]. The details of these studies have already been published in several papers [27–29, 31, 34–36]. All study participants gave written informed consent prior to study participation. These studies were approved by the ethics committees of all the participating centers [30] and were conducted in accordance with “The Code of Ethics of the World Medical Association” [29, 31, 37].

### Data collection and determination of the fit for the aged (FORTA) score

To both include as many older patients (82 years and above) as possible and to assess medication quality as close as possible to the date of publication for the current FORTA list [38], baseline data for this study were collected at follow-up 6; they included drug use (ATC codes), gender, age, GP diagnoses (details are provided below) and blood pressure to determine medication quality according to FORTA as reflected by the FORTA score. This score is the sum of medication errors classified as over- and/or undertreatment errors in an individual patient when checked against the labels in the FORTA list. An error was counted by the investigator if an indication was not appropriately treated though beneficial options (FORTA A or B) exist (undertreatment) or if a prescription was suboptimal regarding the FORTA categories (e.g., FORTA C though A or B drugs exist) or not indicated (over-treatment) [38]. The FORTA score is the sum of over- and undertreatment errors, e.g., overtreatment by a drug which is not indicated or a better one would be available, or undertreatment if a positively indicated drug is not given. Proton-pump-inhibitors are often not indicated and would generate an overtreatment error; oral anticoagulation is strictly indicated in atrial fibrillation, and the absence of a positively labeled oral anticoagulant (e.g., apixaban) would generate an undertreatment error. Further details on the determination of the FORTA score are provided elsewhere [16, 25, 39]. A comprehensive “instruction” for the use of the FORTA list [39] is the prerequisite for the determination of the FORTA score as provided in the original VALFORTA trial [25].

Based on data from follow-up 6, the following alignments were made:

- Gastritis, reflux gastritis, reflux, esophageal carcinoma, and gastrointestinal bleeding were considered to reflect the FORTA diagnosis “gastrointestinal disease.” Lipid metabolism disorder, coronary artery disease (CHD), peripheral arterial occlusive disease, and myocardial infarction (MI) were aligned to the FORTA diagnosis “chronic therapy after myocardial infarction.” Osteoarthritis, chronic back pain, pain syndrome, and fracture were aligned to the FORTA diagnosis “chronic pain.” The diagnoses cardiac arrhythmia, sick sinus syndrome, and atrial fibrillation were aligned as the FORTA diagnosis “atrial fibrillation.” Stroke, cerebellar infarction, stenosis of the afferent cerebral arteries, and transient ischemic attacks were considered the FORTA diagnosis “stroke.” Abnormal behavior was interpreted as the FORTA diagnosis “dementia-associated behavioral problems” if the patient was also diagnosed with dementia. Hypothyroidism, thyroidectomy, and nodular goiter were subordinated under the FORTA diagnosis “hypothyroidism.” COPD and emphysema were aligned to the FORTA diagnosis “chronic obstructive pulmonary disease (COPD).” If there was a diagnosis of arterial hypertension and/or if the systolic blood pressure was above 140 mmHg and/or the diastolic blood pressure was above 85 mmHg, this was considered the FORTA diagnosis “arterial hypertension.” Anemia, iron deficiency anemia, and vitamin B12 deficiency anemia were aligned to the FORTA diagnosis “anemia.” Parkinson’s disease and restless legs were interpreted as the FORTA diagnosis “Parkinson’s disease.”
  The diagnoses diabetes mellitus type 2, dementia, urinary incontinence, infections, epilepsy, depression, osteoporosis, heart failure, and sleep disorders (insomnia) were identical to the original FORTA diagnoses.
  Besides, the severity (numerical grades usually ranged from mild to severe) of some diagnoses following a prespecified protocol were quantified as follows:
- Grade 1–4 heart failure. Grade 1–3 arterial hypertension. Grade 1–4 atrial fibrillation. Grade 1–3 depression. Grade 1–3 osteoporosis. Grade 1–4 dementia. Grade 1–2 hypothyroidism. Grade 1 anemia. Grade 2 obstructive pulmonary disease. Grade 1–2 sleep disorder. Parkinson’s disease grade 1–4. Grade 1–3 urinary incontinence. Grade 3 pain. If the diagnosis of beginning dementia was available, grade 1 was assigned to dementia.

Finally, for the longitudinal analysis, data regarding activities of daily living (ADL) measured by the Barthel Index [40] which is an ordinal scale and includes ten basic activities of self-care, instrumental activities of daily living (IADL) assessed by the Lawton and Brody Scale which covers daily activities beyond self-care [41] and mortality from the follow-up visits 6–9 were used. Since ADL addresses basic activities of daily living such as eating or dressing only, we also used IADL to cover more complex activities of daily living such as managing finances or medications which require higher level of cognitive function.

### Statistical analysis and strengthening the reporting of observational studies in epidemiology (STROBE) [42] statement

Associations between the FORTA score and ADL/IADL were analyzed by Spearman univariate correlation. Statistical comparisons of ADL or IADL for participants with a low FORTA score (equal or below the median obtained for all participants at follow-up 6, FORTA score < 6) vs. patients with a high FORTA score (FORTA score ≥ 6) were performed by the Wilcoxon rank-sum test. The cutoff at 6 (the median) was chosen to provide nearly equal numbers of observations in both groups. The Wilcoxon rank-sum test was also used for comparison of mean FORTA scores of participants with dementia vs. those without dementia and comparison of mean FORTA scores of participants who died vs. those alive. Multivariate correlations between FORTA score/possible confounders (number of medications, gender, age, number of diseases) at follow-up 6 and ADL/IADL at follow-up 6 were performed using the Poisson regression. In addition, the chi-square test was used to assess nominal variables with two categories. Statistical significance was assumed at *p* < 0.05. The STROBE checklist (Supplementary Material 1) was followed to ascertain that relevant data were included.

## Results

A total of 723 participants from follow-up 6 of the AgeCoDe study who had at least five or more long-term medications (i.e., those with polypharmacy according to its definition in the majority of studies [2]) were screened for inclusion. Two hundred nineteen of them were not included as data on GP diagnoses for the determination of the FORTA score were not available. Hence, the FORTA score at follow-up 6 was determined for a total of 504 individuals. The baseline characteristics of these patients such as the mean FORTA score, mean age, mean number of diseases, and the prevalence of the most frequent diseases at follow-up 6 are provided in Table 1.

**Table 1:** The baseline characteristics of the cohort (total number = 504) at follow-up 6. *n* number of cases

**Items**	
Mean FORTA score (median; range)	6.2 (6; 0–18)
Mean age (median; range)	87.9 (87.5; 83–101)
Mean number of diseases (median; range)	5.3 (5; 0–14)
Mean number of medications (median; range)	7.99 (7; 5–23)
Gender female % (*n*)	69.6 (351)
Hypertension % (*n*)	82.3 (415)
Arthritis % (*n*)	61.1 (308)
Lipid metabolism disorder % (*n*)	57.3 (289)
Chronic backpain % (*n*)	42.8 (216)
Coronary heart disease % (*n*)	41.8 (211)
Heart failure % (*n*)	36.7 (185)
Cardiac arrhythmias % (*n*)	35.3 (178)
Type II Diabetes % (*n*)	29.9 (151)
Depression % (*n*)	28.9 (146)
Dementia % (*n*)	17.8 (90)

The univariate analysis of the association between the FORTA score at follow-up 6 and ADL at follow-ups 6–9 showed a significant negative correlation at all follow-ups meaning that higher scores were associated with worse ADL values (Supplementary Material 2).

**Fig. 1:**
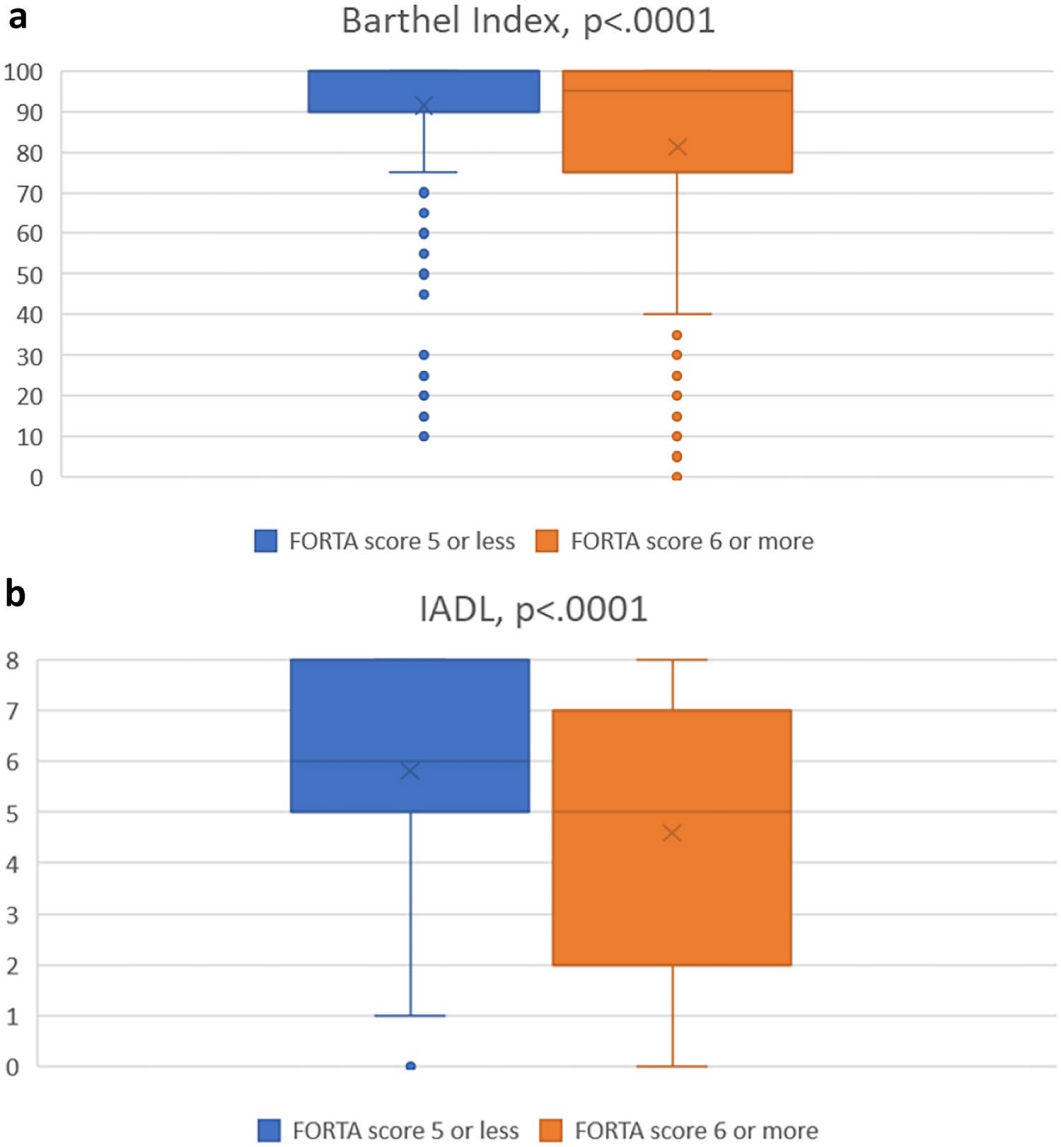
**a**, **b** Box plot of assessment of activities of daily living (Barthel Index) in **a** and instrumental activities of daily living (IADL) according to Lawton and Brody in **b** for the group with low vs. high Fit fOR The Aged (FORTA) scores at follow-up 6; the cutoff at 6 (= median) was chosen to provide nearly equal numbers of observations in both groups. The horizontal line represents the median, the box represents interquartile range, whiskers represent 95% confidence intervals, crosses represent the mean, and the circle represents an outlier

The comparison of ADL measured by the Barthel Index for patients with low vs. high FORTA scores at follow-up 6 also revealed that participants with a higher FORTA score (above the median) had significantly lower ADL scores as compared to those with a lower FORTA score (Fig. 1a).

The univariate analysis of the association between the FORTA score at follow-up 6 and IADL according to Lawton and Brody at follow-ups 6–9 showed a significant negative correlation at all follow-ups as well (Supplementary Material 3).

In addition, the comparison of the IADL results for participants with low vs. high FORTA scores at follow-up 6 also revealed that participants with a higher FORTA score (above the median) had significantly lower IADL scores as compared to those with a lower FORTA score (Fig. 1b).

The significant associations between the FORTA score and ADL or IADL were also confirmed by the multivariate Poisson regression analysis at follow-up 6 (Table 2).

**Table 2:** Multivariate Poisson regression analysis between FORTA score/possible confounders at follow-up 6 and ADL/IADL at follow-up 6. Regression coefficients are provided in parenthesis

	**ADL**	**IADL**
FORTA score	< 0.0001 (− 0.0035)	< 0.0001 (− 0.0337)
Number of medications	< 0.0001 (0.0391)	< 0.0001 (0.0402)
Gender	0.0775 (− 0.077)	0.0104 (− 0.1022)
Age	0.3102 (0.0062)	0.4274 (0.0049)
Number of diseases	0.0141 (0.0156)	0.0216 (0.0145)

In this model, we adjusted for the number of medications, gender, age and the number of diseases. In addition, there were significant associations between the number of diseases or medications and ADL as well as IADL. Gender was also significantly associated with IADL but not with ADL. Correlation coefficients were negative for FORTA score and gender, and positive for number of medications, age and number of diseases.

We also compared the mean FORTA score of participants with dementia with those without dementia at follow-ups 6–9. The mean FORTA score of participants with dementia was significantly higher than in those without dementia at all follow-ups (Table 3).

**Table 3:** Comparison of mean FORTA scores of participants who died vs. those alive (right) and comparison of mean FORTA scores of participants with dementia vs. those without dementia (left, Wilcoxon-Mann–Whitney test); *n* number of cases, *FU* follow-up

	**Mean FORTA score of participants with dementia (*n*)**	**Mean FORTA score of participants without dementia (*n*)**	***p* value**	**Mean FORTA score of all participants who died since FU6 (*n*)**	**Mean FORTA score of all participants still alive since FU6 (*n*)**	***p* value**
FU6	7.52 (90)	5.77 (414)	< 0.0001	-	-	-
FU7	7.31 (75)	5.72 (374)	< 0.0001	6.98 (55)	6.06 (449)	0.0154
FU8	7.16 (67)	5.72 (360)	< 0.0001	7.08 (77)	5.99 (427)	0.0007
FU9	6.93 (55)	5.71 (340)	< 0.0001	6.86 (109)	5.96 (395)	0.0006

Further comparisons regarding the presence of dementia in participants with low vs. high FORTA score at follow-up 6 revealed that dementia was significantly (*p* < 0.01) more frequent in patients with higher FORTA scores than in those with lower FORTA scores.

Finally, we compared the mean FORTA score of participants who died after follow-up 6 with those who were alive at follow-ups 7, 8 or 9. The mean FORTA scores of participants who died were significantly higher than those in survivors at follow-up 7, 8 or 9 (Table 3).

## Discussion

Our findings show significant correlations between medication quality and functional status in community-dwelling older adults. Although weak according to Leclezio et al. [43], these correlations are in line with our previous findings from the VALFORTA trial [16, 25] and with those reported in the literature [11]. While ADL and IADL were negatively correlated with the FORTA score both longitudinally as well as in the multivariate analysis thus matching with those studies cited, they were positively correlated with the number of medications and diseases. This could mean that more positively labeled drugs may have been given to sicker patients resulting in this positive correlation, but this explanation remains speculative.

The potential associative link between inappropriate prescribing and dementia as well as mortality shown here has also been described in other studies [7, 9, 10, 12–15]. While numerous studies on the association between polypharmacy and adverse outcomes in older adults exist, only few interventional studies on the impact of inappropriate prescribing on clinical outcomes in older adults have been performed [2, 21, 44, 45]. One study using the STOPP/START criteria in nursing home residents did not show a significant difference between the control and intervention group regarding the impact on functional status assessed by the Functional Independence Measure (FIM) [46]. Similarly, interventional trials assessing the impact of inappropriate prescribing on dementia and mortality are scarce [21]. So far, only one study showed a positive impact of drug optimization (combining the Medication Appropriateness Index (MAI), Beers Criteria®, and the “underutilization of medication instrument”) on serious adverse drug reactions including mortality in frail elderly patients [47].

In a previous association study, we found that higher FORTA scores indicating more frequent medication errors are associated with impaired cognitive and physical function tests in older hospitalized patients [16]. In addition, activities of daily living (ADL) were significantly improved through the FORTA intervention in the VALFORTA trial [25]. Due to the short period of hospitalization and, thus, intervention in this trial, some adverse clinical outcomes of inappropriate prescribing such as mortality or cognitive incapacity may have not been detectable. Therefore, a longer observation period as provided in the present study was required to examine possible correlations for these outcomes. Here, we were able to demonstrate an association between higher FORTA scores and lower ADL as well as IADL values in older community-dwelling adults. In addition, higher FORTA scores appear to be linked to a higher incidence of dementia and even mortality. The results from a previous study by Heser et al. [48] which showed that the intake of potentially inappropriate antidepressants is associated with an increased subsequent risk of dementia, are also in line with our findings; those antidepressants are negatively labeled in the FORTA list and are likely to have contributed to higher FORTA scores.

As a major objective of the cohort study analyzed here, the incidence of dementia was determined longitudinally. In this context, it is of paramount importance to find optimal medication schemes not resulting in the prolonged impairment of cognitive function that could qualify as drug-induced dementia [10, 49]; this form of dementia is the most frequent form of reversible dementia and detoxification is the appropriate measure for improvement [50]. The association of dementia and higher FORTA scores in this study might show a significant contribution of drug-induced dementia to the overall burden of dementia [10]. Nevertheless, these noninterventional, observational data do not allow to draw any conclusions on a causal relationship. An increased use of psychoactive drugs resulting in higher FORTA scores may simply reflect an increased burden of treatable CNS disorders, and thus mirror the fact that these patients were sicker than those with lower scores.

The same reflections on causality assessment hold true for the association of the FORTA score and mortality; looking at just one of the many CNS-depressant drug groups, sedating antihistamines, this group of drugs was found to almost double mortality mostly reflecting higher fall and fracture rates [51]. In turn, more morbid patients may be reactively exposed to more toxic drugs for which no better alternatives exist, but still die from those underlying diseases rather than drugs. A more detailed analysis of individual drugs/drug groups and these correlations in the current study is underway.

Yet, the associations between medication quality as determined by the FORTA score and functional outcomes and even mortality point to the importance of balancing increasing medical needs in older patients with detrimental noxious effects of multiple medications. These associations originally found and interventionally corroborated for causality in hospitalized patients have now been confirmed for the much larger group of home-dwelling older people for the first time, though the lack of an intervention does not allow for causality assessment in this group.

### Limitations

In contrast to VALFORTA, the determination of the FORTA score here was limited to available GP based diagnoses which did not include all FORTA relevant diseases and was not supported by full patient records. Moreover, 219 patients were not included as data on GP diagnoses and thus a key element for the determination of the FORTA score were not available. However, this group was not significantly different to the 504 patients included regarding age, gender and number of medications. In addition, the alignment of some diagnoses to one FORTA diagnosis may appear to be arbitrary, and other alignments could be discussed that would have affected the analysis. In other words, not all diagnoses covered by the FORTA list were recorded in this study, but some available diagnoses appeared to be close to those missing, and were therefore aligned to original FORTA diagnoses to increase the information yield. This alignment, however, may have caused bias and other alignments could be discussed that would have affected the final analysis. Hence, the FORTA scores used in our analysis are only estimates of the “real” FORTA scores that could not be determined without knowing more details of the patients and having access to the full patient records. Moreover, after its initial determination, alterations in the FORTA scores after follow-up 6 cannot be excluded as the diagnoses and pharmacotherapy of the participants may have been subject to changes. Thus, the associations with ADL/IADL as well as correlations with the incidence of dementia and mortality shown here do not reflect adjustments of the FORTA score at later visits which in turn could have influenced these correlations.

Finally, the findings of this study are limited to the determination of associations between the quality of medication and the aforementioned outcomes. The results do not prove causality, and hypotheses derived from them here need to be validated in randomized controlled trials.

## Conclusions and implications

Higher FORTA scores are associated with worse functional outcomes in older community-dwelling adults. Therefore, this score may predict important patient-relevant outcomes and could become a useful tool to scrutinize pharmacological treatment in older people with the aim of improving essential outcomes.

## Supplementary information

## Supplementary Information

Below is the link to the electronic supplementary material.

Supplementary file1 (DOCX 36 KB)

Supplementary file2 (DOCX 16 KB)

Supplementary file3 (DOCX 16 KB)
