# Supplementary material for: Higher FORTA (Fit fOR The Aged) scores are associated with poor functional outcomes, dementia, and mortality in older people
Source: Eur J Clin Pharmacol. 2022 Sep 27;78(11):1851–9. doi: 10.1007/s00228-022-03389-w (PMC9546968; doi:10.1007/s00228-022-03389-w)
Supplement: Supplementary file 1 — Supplementary file1 (DOCX 36 KB) [file 228_2022_3389_MOESM1_ESM.docx]

*European Journal of Clinical Pharmacology*

**Supplementary Material 3**

**Article Title: Higher FORTA (Fit fOR The Aged) Scores are Associated with poor Functional Outcomes, Dementia and Mortality in Older People**

**Authors names:** Farhad Pazan^1^, Hanna Breunig^1^, Christel Weiss^2^, Susanne Röhr^3^, Melanie Luppa^3^, Michael Pentzek^4^, Horst Bickel^5^, Dagmar Weeg^5^, Siegfried Weyerer^6^, Birgitt Wiese^7^, Hans-Helmut König^8^, Christian Brettschneider^8^, Kathrin Heser^9^, Wolfgang Maier^9^, Martin Scherer^10^, Steffi Riedel-Heller^3^, Michael Wagner^9, 11^ **&**, Martin Wehling^1^**&**

**&** Shared Last Authorship

1 Clinical Pharmacology Mannheim, Medical Faculty Mannheim, Ruprecht-Karls-Heidelberg University, Theodor-Kutzer-Ufer 1-3, 68167, Mannheim, Germany.

2 Department of Medical Statistics, Biomathematics and Information Processing, Medical Faculty of the University of Heidelberg in Mannheim, Germany.

3 Institute of Social Medicine, Occupational Health and Public Health (ISAP), Medical Faculty, University of Leipzig, Germany.

4 Institute of General Practice, Medical Faculty, Heinrich-Heine-University Düsseldorf, Germany

5 Department of Psychiatry, Technical University of Munich, Germany.

6 Central Institute of Mental Health, Medical Faculty Mannheim/Heidelberg University, Mannheim, Germany.

7 Institute for General Practice, Hannover Medical School, Germany.

8 Department of Health Economics and Health Services Research, University Medical Centre Hamburg-Eppendorf, Germany.

9 Department of Neurodegenerative Diseases and Geriatric Psychiatry, University Hospital Bonn, Bonn, Germany.

10 Department of Primary Medical Care, Center for Psychosocial Medicine, University Medical Center Hamburg-Eppendorf, Germany.

11 German Center for Neurodegenerative Diseases (DZNE), Bonn, Germany

Corresponding author:

Prof. Dr. med. Martin Wehling

Clinical Pharmacology Mannheim

Medical Faculty Mannheim

Ruprecht-Karls-University Heidelberg

Theodor-Kutzer-Ufer 1-3

68167 Mannheim

Germany

E-Mail: [martin.wehling@medma.uni-heidelberg.de](mailto:martin.wehling@medma.uni-heidelberg.de)

| **IADL** | **Number of Patients** | **Median/IQR for IADL** | **Spearman correlation coefficient** | **p value** |
| --- | --- | --- | --- | --- |
| **FU6** | 504 | 6/5 | -0.243 | < 0.0001 |
| **FU7** | 399 | 5/5 | -0.268 | < 0.0001 |
| **FU8** | 345 | 5/4 | -0.271 | < 0.0001 |
| **FU9** | 288 | 5/5 | -0.239 | < 0.0001 |

**Supplementary Material 3** Association between the FORTA score at follow-up 6 and Instrumental activities of daily living (IADL) according to Lawton and Brody at follow-up 6-9. FU: follow-up. IQR: Interquartile Range (Q3-Q1)
