# Supplementary material for: Higher FORTA (Fit fOR The Aged) scores are associated with poor functional outcomes, dementia, and mortality in older people
Source: Eur J Clin Pharmacol. 2022 Sep 27;78(11):1851–9. doi: 10.1007/s00228-022-03389-w (PMC9546968; doi:10.1007/s00228-022-03389-w)
Supplement: Supplementary file 2 — Supplementary file2 (DOCX 16 KB) [file 228_2022_3389_MOESM2_ESM.docx]

*European Journal of Clinical Pharmacology*

**Supplementary Material 1**

**Article Title: Higher FORTA (Fit fOR The Aged) Scores are Associated with poor Functional Outcomes, Dementia and Mortality in Older People**

**Authors names:** Farhad Pazan^1^, Hanna Breunig^1^, Christel Weiss^2^, Susanne Röhr^3^, Melanie Luppa^3^, Michael Pentzek^4^, Horst Bickel^5^, Dagmar Weeg^5^, Siegfried Weyerer^6^, Birgitt Wiese^7^, Hans-Helmut König^8^, Christian Brettschneider^8^, Kathrin Heser^9^, Wolfgang Maier^9^, Martin Scherer^10^, Steffi Riedel-Heller^3^, Michael Wagner^9, 11^ **&**, Martin Wehling^1^**&**

**&** Shared Last Authorship

1 Clinical Pharmacology Mannheim, Medical Faculty Mannheim, Ruprecht-Karls-Heidelberg University, Theodor-Kutzer-Ufer 1-3, 68167, Mannheim, Germany.

2 Department of Medical Statistics, Biomathematics and Information Processing, Medical Faculty of the University of Heidelberg in Mannheim, Germany.

3 Institute of Social Medicine, Occupational Health and Public Health (ISAP), Medical Faculty, University of Leipzig, Germany.

4 Institute of General Practice, Medical Faculty, Heinrich-Heine-University Düsseldorf, Germany

5 Department of Psychiatry, Technical University of Munich, Germany.

6 Central Institute of Mental Health, Medical Faculty Mannheim/Heidelberg University, Mannheim, Germany.

7 Institute for General Practice, Hannover Medical School, Germany.

8 Department of Health Economics and Health Services Research, University Medical Centre Hamburg-Eppendorf, Germany.

9 Department of Neurodegenerative Diseases and Geriatric Psychiatry, University Hospital Bonn, Bonn, Germany.

10 Department of Primary Medical Care, Center for Psychosocial Medicine, University Medical Center Hamburg-Eppendorf, Germany.

11 German Center for Neurodegenerative Diseases (DZNE), Bonn, Germany

Corresponding author:

Prof. Dr. med. Martin Wehling

Clinical Pharmacology Mannheim

Medical Faculty Mannheim

Ruprecht-Karls-University Heidelberg

Theodor-Kutzer-Ufer 1-3

68167 Mannheim

Germany

E-Mail: [martin.wehling@medma.uni-heidelberg.de](mailto:martin.wehling@medma.uni-heidelberg.de)

STROBE Statement—checklist of items that should be included in reports of observational studies

|  | Item No | Recommendation |
| --- | --- | --- |
| **Title and abstract** | 1 | (*a*) Indicate the study’s design with a commonly used term in the title or the abstract  See page 1 |
|  |  | (*b*) Provide in the abstract an informative and balanced summary of what was done and what was found  See page 1 |
| Introduction | | |
| Background/rationale | 2 | Explain the scientific background and rationale for the investigation being reported  See page 2 |
| Objectives | 3 | State specific objectives, including any prespecified hypotheses  See page 2 (4^th^ paragraph) |
| Methods | | |
| Study design | 4 | Present key elements of study design early in the paper  See page 3 (“2.1 Study design”) |
| Setting | 5 | Describe the setting, locations, and relevant dates, including periods of recruitment, exposure, follow-up, and data collection  See page 3 (“2.1 Study design”) |
| Participants | 6 | (*a*) *Cohort study*—Give the eligibility criteria, and the sources and methods of selection of participants. Describe methods of follow-up  See page 3 (“2.1 Study design”)  *Case-control study*—Give the eligibility criteria, and the sources and methods of case ascertainment and control selection. Give the rationale for the choice of cases and controls  *Cross-sectional study*—Give the eligibility criteria, and the sources and methods of selection of participants |
|  |  | (*b*) *Cohort study*—For matched studies, give matching criteria and number of exposed and unexposed N/A  *Case-control study*—For matched studies, give matching criteria and the number of controls per case |
| Variables | 7 | Clearly define all outcomes, exposures, predictors, potential confounders, and effect modifiers. Give diagnostic criteria, if applicable  See page 3 and 4 (“2.2 Data collection & determination of the Fit fOR The Aged (FORTA) score”) |
| Data sources/ measurement | 8* | For each variable of interest, give sources of data and details of methods of assessment (measurement). Describe comparability of assessment methods if there is more than one group  See page 3 (“2.2 Data collection & determination of the Fit fOR The Aged (FORTA) score”) |
| Bias | 9 | Describe any efforts to address potential sources of bias  See page 9 (second paragraph) |
| Study size | 10 | Explain how the study size was arrived at  See page 2-3 |
| Quantitative variables | 11 | Explain how quantitative variables were handled in the analyses. If applicable, describe which groupings were chosen and why  See page 3-4 |
| Statistical methods | 12 | (*a*) Describe all statistical methods, including those used to control for confounding  See page 4 (“Statistical analysis & STrengthening the Reporting of OBservational studies in Epidemiology (STROBE) [42] statement”) |
|  |  | (*b*) Describe any methods used to examine subgroups and interactions  See page 4 (“Statistical analysis & STrengthening the Reporting of OBservational studies in Epidemiology (STROBE) [42] statement”) |
|  |  | (*c*) Explain how missing data were addressed  See page 4 and 9 |
|  |  | (*d*) *Cohort study*—If applicable, explain how loss to follow-up was addressed  *Case-control study*—If applicable, explain how matching of cases and controls was addressed  *Cross-sectional study*—If applicable, describe analytical methods taking account of sampling strategy |
|  |  | (*e*) Describe any sensitivity analyses |

Continued on next page

| Results | | |
| --- | --- | --- |
| Participants | 13* | (a) Report numbers of individuals at each stage of study—eg numbers potentially eligible, examined for eligibility, confirmed eligible, included in the study, completing follow-up, and analysed  See page 4 (“Results”) |
|  |  | (b) Give reasons for non-participation at each stage  See page 4 and 9 |
|  |  | (c) Consider use of a flow diagram  For space reasons, we refer to previous publications of our cohort. |
| Descriptive data | 14* | (a) Give characteristics of study participants (eg demographic, clinical, social) and information on exposures and potential confounders  See page 4-5 |
|  |  | (b) Indicate number of participants with missing data for each variable of interest  See page 4-9 |
|  |  | (c) *Cohort study*—Summarise follow-up time (eg, average and total amount)  See detailed description on page 3 |
| Outcome data | 15* | *Cohort study*—Report numbers of outcome events or summary measures over time  See page 4-7 |
|  |  | *Case-control study—*Report numbers in each exposure category, or summary measures of exposure |
|  |  | *Cross-sectional study—*Report numbers of outcome events or summary measures |
| Main results | 16 | (*a*) Give unadjusted estimates and, if applicable, confounder-adjusted estimates and their precision (eg, 95% confidence interval). Make clear which confounders were adjusted for and why they were included  See page 4-7 |
|  |  | (*b*) Report category boundaries when continuous variables were categorized  See page 4-7 |
|  |  | (*c*) If relevant, consider translating estimates of relative risk into absolute risk for a meaningful time period |
| Other analyses | 17 | Report other analyses done—eg analyses of subgroups and interactions, and sensitivity analyses |
| Discussion | | |
| Key results | 18 | Summarise key results with reference to study objectives  See page 7-9 |
| Limitations | 19 | Discuss limitations of the study, taking into account sources of potential bias or imprecision. Discuss both direction and magnitude of any potential bias  See page 9 (“Limitations”) |
| Interpretation | 20 | Give a cautious overall interpretation of results considering objectives, limitations, multiplicity of analyses, results from similar studies, and other relevant evidence  See page 7-9 |
| Generalisability | 21 | Discuss the generalisability (external validity) of the study results  See page 9 |
| Other information | | |
| Funding | 22 | Give the source of funding and the role of the funders for the present study and, if applicable, for the original study on which the present article is based  See page 9 (“Funding”) |

*Give information separately for cases and controls in case-control studies and, if applicable, for exposed and unexposed groups in cohort and cross-sectional studies.

**Note:** An Explanation and Elaboration article discusses each checklist item and gives methodological background and published examples of transparent reporting. The STROBE checklist is best used in conjunction with this article (freely available on the Web sites of PLoS Medicine at http://www.plosmedicine.org/, Annals of Internal Medicine at http://www.annals.org/, and Epidemiology at http://www.epidem.com/). Information on the STROBE Initiative is available at www.strobe-statement.org.
